# Serum neurofilament light levels in normal aging and their association with morphologic brain changes

**DOI:** 10.1038/s41467-020-14612-6

**Published:** 2020-02-10

**Authors:** Michael Khalil, Lukas Pirpamer, Edith Hofer, Margarete M. Voortman, Christian Barro, David Leppert, Pascal Benkert, Stefan Ropele, Christian Enzinger, Franz Fazekas, Reinhold Schmidt, Jens Kuhle

**Affiliations:** 1 Department of Neurology, Medical University of Graz, Auenbruggerplatz 22, 8036 Graz, Austria; 2 Institute for Medical Informatics, Statistics and Documentation, Medical University of Graz, Auenbruggerplatz 2, 8036 Graz, Austria; 3 Neurologic Clinic and Policlinic, Departments of Medicine, Biomedicine and Clinical Research, University Hospital Basel, University of Basel, Petersgraben 4, 4031 Basel, Switzerland; 4 Clinical Trial Unit, Department of Clinical Research, University Hospital Basel, University of Basel, Spitalstrasse 21, 4031 Basel, Switzerland; 5 Division of Neuroradiology, Vascular and Interventional Radiology, Department of Radiology, Medical University of Graz, Auenbruggerplatz 9, 8036 Graz, Austria

**Keywords:** Biomarkers, Neurological disorders

## Abstract

Neurofilament light (NfL) protein is a marker of neuro-axonal damage and can be measured not only in cerebrospinal fluid but also in serum, which allows for repeated assessments. There is still limited knowledge regarding the association of serum NfL (sNfL) with age and subclinical morphologic brain changes and their dynamics in the normal population. We measured sNfL by a single molecule array (Simoa) assay in 335 individuals participating in a population-based cohort study and after a mean follow-up time of 5.9 years (n = 103). Detailed clinical examination, cognitive testing and 3T brain MRI were performed to assess subclinical brain damage. We show that rising and more variable sNfL in individuals >60 years indicate an acceleration of neuronal injury at higher age, which may be driven by subclinical comorbid pathologies. This is supported by a close association of sNfL with brain volume changes in a cross-sectional and especially longitudinal manner.

## Abstract (web summary)

Neurofilament (NfL) levels in CSF and blood have been established as a biomarker of neuronal damage in neurodegenerative diseases, and there is an age-dependent increase in NfL levels in CSF. Here the authors demonstrate that serum NfL levels increase in healthy aging people and predict and correlate with brain volume loss.

## Introduction

Neuro-axonal damage is a consequence of many neurologic diseases and contributes to deficits and disability. For research and clinical practice, it would therefore be of eminent importance to have a tool that can reliably detect and monitor such kind of damage^1^. In the last few years, neurofilament (Nf) proteins have gained increasing attention in this direction. Neuro-axonal damage causes their release into the extracellular space and further into the cerebrospinal fluid (CSF) and the blood and Nf may therefore provide real-time information about neuro-axonal injury in the central nervous system (CNS)^1^. Until recently Nf studies were limited to CSF, because detection systems were not sensitive enough to quantitate the physiologically lower levels of Nf in the peripheral blood and this restricted clinical applicability. Conversely, to obtain CSF requires lumbar puncture, which is an invasive procedure^2^, requiring stringent indication for diagnostic purpose. Repeated CSF collection, i.e. follow-up studies are even more difficult to justify. This has changed with the introduction of the single molecule array (Simoa) technology^3^, which provides now the analytical basis for highly sensitive quantitation of the Nf light (NfL) subunit in the peripheral blood^4,5^. Of note, several studies have demonstrated that CSF and serum NfL (sNfL) levels are highly correlated and this has given reason to study sNfL in a wide range of neurologic disorders, including some in which a lumbar puncture would not be performed for diagnostic purposes^1^.

In order to correctly interpret sNfL levels in disease states, it is essential to know if and how the concentration of this protein changes with age and gender in neurologically inconspicuous individuals. Furthermore, the association between sNfL levels and subclinical morphologic brain changes appears quite important as these are frequently seen during the course of normal aging^6,7^. We therefore investigated sNfL in a single-center cohort of 335 participants (age range 38.5−85.6 years) of the Austrian Stroke Prevention Study^8,9^, which is a prospective community-based study on brain health and aging in Graz, Austria. We hypothesized that the sNfL levels would increase with aging and levels would vary more strongly with age due to an increasing frequency of concomitant subclinical cerebral pathology.

We show that rising and more variable sNfL levels in individuals >60 years indicates an acceleration of neuronal injury at higher age, which may be driven by subclinical comorbid pathologies. This is supported by a close association of sNfL concentrations with brain volume changes in a cross-sectional and especially longitudinal manner.

## Results

The dataset consisted of 335 (195 females/140 males) neurologically inconspicuous community-dwelling individuals. Their age ranged from 38.5 to 85.6 years with a mean age of 64.9 (standard deviation (SD) = ± 10.8) years. In 103 subjects (47 females/56 males), a follow-up visit had been performed after a mean of 5.9 (SD = ± 1.0) (min = 4.0, max = 6.9) years, including serum sampling and MRI. Table 1 displays the demographic characteristics, vascular risk factors, lifestyle factors and MRI metrics of the study cohort. The median (IQR) sNfL values of the cohort were 32.30 pg mL^−1^ (23.15−43.95) and the median annualized sNfL change 3.22% (0.91−6.73%) (Table 1).

**Table 1:** Descriptive statistics of demographics, morphometric data, risk factors and lifestyle factors of the examined population.

Demographics				
	Mean age (years)^a^		64.86	(10.76)
	*N* subjects (female, male)		335	(195, 140)
Risk factors				
	Hypertension or hypertension history (%)^a^		62.42	(48.51)
	Systolic blood pressure (mmHg)^b^		136	(120−150)
	Diastolic blood pressure (mmHg)^b^		85	(80−91)
	Hypercholesterolemia (%)^a^		75.47	(43.10)
	Cholesterol (mg dL^−1^)^b^		208.5	(182−235)
	High-density lipoprotein (mg dL^−1^)^b^		66	(54−80)
	Low-density lipoprotein (mg dL^−1^)^b^		117	(96−141)
	Diabetes (%)^a^		8.06	(27.26)
	Glycated hemoglobin (mg dL^−1^)^b^		5.50	(5.30−5.80)
	Blood sugar level (mg dL^−1^)^b^		93.00	(86.25−102.00)
Lifestyle				
	Smoking (%)^a^		15.82	(36.55)
	Alcohol (number of glasses)^a^		1.27	(4.50)
	BMI (kg m^−2^)^b^		25.67	(23.56−28.73)
	Waist-hip ratio (%)^b^		87.78	(81.62−93.20)
MRI				
	Brain volume (cm^3^)^a^		1495.65	(80.52)
	Brain atrophy annualized (%)^a,c^		−0.58	(0.27)
	WMH volume (cm^3^)^a^		7.12	(11.01)
	WMH volume change annualized (%)^b^		8.54	(3.15−16.65)
Serum neurofilament light				
	sNfL (pg mL^−1^)^b^		32.30	(23.15−43.95)
	sNfL change annualized (%)^b,c^		3.22	(0.91−6.73)

^a^Mean (standard deviation).

^b^Median (25th−75th percentile).

^c^Only subjects with BL and FU data, *N* = 95.

### Association of sNfL with age and gender

**Fig. 1:**
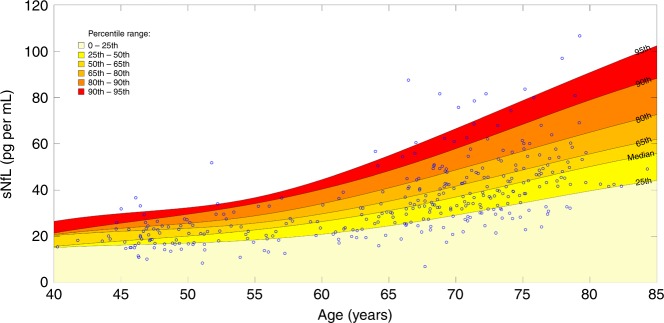
Percentile ranges of sNfL. Percentile range areas of sNfL during the course of normal aging: The spline interpolated PCTL range curves are based on the sNfL measures in each age category (see Table 2 and Table 3). Source data are provided as a Source Data file.

**Fig. 2:**
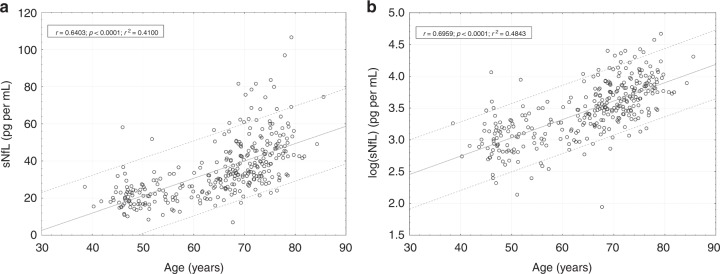
Correlation of sNfL with age. Scatterplot of sNfL over age (**a**) and logarithmic transformed sNfL values (**b**). The dashed lines represent the 90% prediction interval, the continuous line denotes the regression line. (*r* = Pearson regression coefficient, *p* = two-sided *p* value). Source data are provided as a Source Data file.

To explore the relation with age, we calculated sNfL levels in the different decades for the examined population (Tables 2 and 3). As can be seen, median (25th–75th percentile) sNfL (pg mL^−1^) levels were quite similar between the 4th (18.90 (16.70−24.10); *n* = 54) and 5th (22.10 (17.95–26.45); *n* = 45) decades but then increased in a nonlinear manner: 32.40 (25.50–40.70) (60−70 years, *n* = 102); 43.30 (34.20–53.40) (>70 years, *n* = 134). The strong correlation between age and sNfL is shown in Fig. 1 and also in Fig. 2, on untransformed (Fig. 2a, Pearson correlation (*r*_p_) = 0.64, *p* < 0.0001) and on log-transformed sNfL levels (Fig. 2b, *r*_p_ = 0.70, *p* < 0.0001).

**Table 2:** Descriptive statistics of sNfL (pg mL^−1^) levels in examined population per age groups.

Age range	Mean age (SD)	Sex (f/m)	*N*	Mean sNfL (SD)	Median sNfL (IQR)	Min sNfL	Max sNfL
<50	46.66 (2.22)	27/27	54	20.40 (5.61)	18.90 (7.40)	10.2	36.7
50−60	54.16 (2.92)	27/18	45	22.85 (7.68)	22.10 (8.50)	8.5	51.8
60−70	66.37 (2.61)	65/37	102	34.69 (13.09)	32.40 (15.20)	7.0	87.5
>70	74.63 (3.15)	76/58	134	45.85 (15.31)	43.30 (19.20)	21.5	106.6

Source data are provided as a Source Data file.

*SD* standard deviation, *f* female, *m* male, *IQR* interquartile range, *N* number of samples.

**Table 3:** Percentiles and annual increase of sNfL in each age group.

Age range	*N*	25th	60th	70th	75th	80th	85th	90th	95th	Median ΔsNfL p.a. in % (25th 75th PCTL)	*N* ΔsNfL
<50	54	16.70	21.42	23.20	24.10	24.57	26.04	27.23	31.40	0.94% (−1.12% 3.70%)	13
50−60	45	17.95	23.85	25.90	26.45	27.60	29.60	33.00	34.73	2.73% (0.88% 5.75%)	13
60−70	102	25.50	34.58	39.28	40.70	43.05	48.38	50.50	58.22	4.32% (1.69% 7.89%)	40
>70	134	34.20	46.55	49.59	53.40	56.34	60.10	67.91	77.94	4.23% (2.02% 6.74%)	29

Source data are provided as a Source Data file.

*ΔsNfL* change of sNfL, *p.a.* per annum, *PCTL* percentile, *N* number of samples, *N ΔsNfL* number of follow-up samples.

There was no gender effect on sNfL when analyzing the entire cohort as well as each age category. We tested whether the sNfL levels were related to the storage time. These measures did not show a significant correlation (Spearman correlation (*r*_s_) = −0.107, *p* = n.s.), in line with findings by others^4,10^. We therefore did not consider this variable in further analysis.

**Fig. 3:**
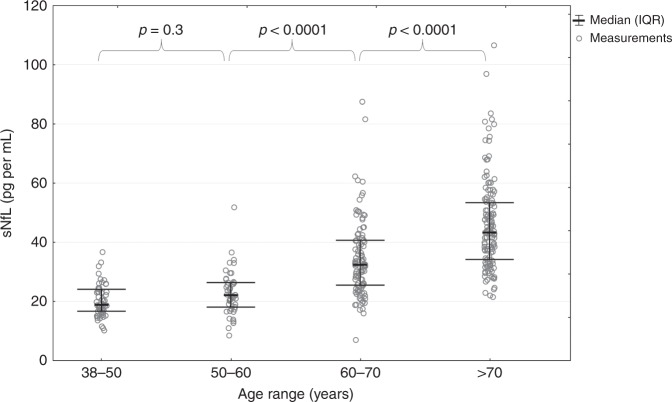
Variance of sNfL per age groups. Variance analysis: original sNfL values for each age category. The *p* values result from the Brown−Forsythe test, which analyzes the equality of the variance in two adjacent age categories. While the variance in the first two categories can be considered as equal, it is increased significantly in the age categories > 60 years. Source data are provided as a Source Data file.

To visualize the development of sNfL levels over the lifespan (Fig. 1), we analyzed the percentiles (PCTLs) in each decade of the examined population graduated by PCTL ranges, which are represented by colored areas. The mean increase of sNfL levels with age was paralleled by an increase of the spread of sNfL values. This increase of variance was statistically significant above 60 years of age (*p* < 0.0001 using Brown−Forsythe test) (Fig. 3).

### Temporal dynamics of sNfL levels

**Fig. 4:**
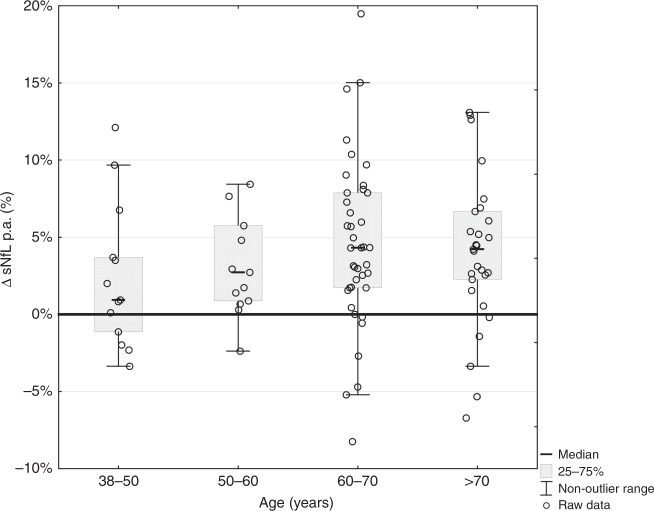
Annualized increase of sNfL per age groups. Annualized increase of sNfL in different age groups. Bars represent median values of proportional annual change per age group. A substantial higher increase is seen in the age groups above 60 years. sNfL serum neurofilament light, p.a. per annum. Source data are provided as a Source Data file.

We then analyzed the subset of 103 individuals with available follow-up sNfL measures. After exclusion of eight cases who became diseased during follow-up, the final longitudinal dataset consisted of 95 individuals. Comparing baseline and follow-up sNfL levels revealed that the yearly median increase was 0.9% in the age group 40−50 years and 2.7% in the age group 50−60 years, reflecting a nonlinear increase. Above 60 years the yearly median increase in sNfL reached a maximum slope of up to 4.3%. In contrast, above 70 years, the slope of annual change in sNfL remained stable at this rate (Table 3, last row and Fig. 4).

Using the suggested progression of the PCTL ranges displayed in Fig. 1, we found that 87.37% of the follow-up sNfL levels were inside the same PCTL range, as previously determined by their baseline sNfL levels. Furthermore, we found a significant increase in the spread of sNfL levels during follow-up (*p* < 0.05 using Brown−Forsythe test), when comparing baseline with follow-up sNfL levels (Fig. 3 and also visualized in Fig. 4).

### Association of sNfL with morphologic data

For evaluating the association of sNfL with MRI data, we first performed statistical analysis on the entire cohort but then analyzed subgroups of individuals < and ≥60 years of age according to the increase and higher variability of sNfL levels around that cut-off.

Univariate analysis of the entire cohort (Table 4, left column) showed that baseline sNfL was correlated with normalized brain volume (*r*_s_ = −0.321, *p* < 0.0001) and white matter hyperintensities (WMH) volume (*r*_s_ = 0.325, *p* < 0.0001) at baseline. Baseline sNfL levels were further correlated with the change in brain volume over time (*r*_s_ = −0.380, *p* < 0.001) but not with the change in WMH volume. The change in sNfL correlated with change in brain volume over time (*r*_s_ = −0.290, *p* < 0.01) but not with longitudinal changes in WMH volumes. After correcting for age by applying Spearman partial correlations, only the correlation of change in sNfL with brain atrophy remained significant.

**Table 4:** Results of univariate analysis applying Spearman partial correlation between sNfL levels and morphologic data, with and without consideration of age effects.

	Entire cohort		Age < 60		Age > 60	
	*r*_s_	*p* value	*r*_s_	*p* value	*r*_s_	*p* value
sNfL at baseline						
	***n*** = **335**		***n*** = **99**		***n*** = **236**	
Normalized brain volume at baseline	−0.321	<0.0001	0.204	n.s.	−0.088	n.s.
WMH volume at baseline	0.325	<0.0001	−0.029	n.s.	0.141	<0.05
MMSE at baseline	−0.170	<0.01	0.112	n.s.	−0.081	n.s.
	***n*** = **95**		***n*** = **26**		***n*** = **69**	
Annualized brain atrophy	−0.380	<0.001	0.156	n.s.	−0.110	n.s.
Annualized WMH volume change	0.186	n.s.	−0.116	n.s.	0.117	n.s.
Annualized MMSE change	−0.273	<0.01*	−0.323	n.s.	−0.187	n.s.
Annualized change of sNfL						
Annualized brain atrophy	−0.290	<0.01*	−0.324	n.s.	−0.149	n.s.
Annualized WMH volume change	−0.004	n.s.	−0.042	n.s.	−0.025	n.s.
Annualized MMSE change	−0.091	n.s.	−0.190	n.s.	−0.047	n.s.

Due to motion artifacts or compliance of the subjects, the number of subjects in following variables was reduced: brain volume (*n* = 330), brain volume change (*n* = 89), WMH volume (*n* = 320), WMH volume change (*n* = 88), MMSE change (*n* = 94). * remains significant after the correction for age. Source data are provided as a Source Data file.

We then performed stepwise linear regression analysis (Table 5, upper row) to identify variables explaining the development of brain atrophy. The regression model included age, normalized brain volume, WMH volume, sNfL at baseline and annualized change of sNfL (for the stepwise regression model sNfL was log-transformed). This analysis revealed baseline sNfL (*β* = −0.344, *p* < 0.01), the annualized change in sNfL (*β* = −0.336, *p* < 0.001) followed by age (*β* = −0.302, *p* < 0.05) as the strongest determinants of brain atrophy.

**Table 5:** Results of the stepwise regression to identify the main determinants of brain atrophy.

Selection	Dependent variable	Independent variable	Beta	95% CI	*N*	*p* value
All	Annualized brain atrophy	log(sNfL at BL)	−0.344	(−0.597, −0.091)	95	*p* < 0.01
		Annualized change of sNfL	−0.336	(−0.528, −0.145)	95	*p* < 0.001
		Age	−0.302	(−0.542, −0.062)	95	*p* < 0.05
<60	Annualized brain atrophy	Normalized brain volume	0.530	(0.164, 0.896)	26	*p* < 0.01
≥60	Annualized brain atrophy	log(sNfL at BL)	−0.463	(−0.727, −0.200)	69	*p* < 0.001
		Annualized change of sNfL	−0.393	(−0.656, −0.130)	69	*p* < 0.01

Included variables were: age, normalized brain volume, WMH volume, log(sNfL at baseline) and annualized change of sNfL. Due to motion artifacts or compliance of the subjects, the number of subjects in following variables was reduced: brain volume (*n* = 330), brain volume change (*n* = 89), WMH volume (*n* = 320). Source data are provided as a Source Data file.

*CI* confidence interval.

In the same way we then analyzed only individuals < 60 years of age. Considering this subgroup, cross-sectional analysis (Table 4, central column) showed no significant correlation between baseline sNfL, normalized brain volume and WMH volume. Baseline sNfL levels were also unrelated to the change in total brain and WMH volume over time. There was a trend that increases in sNfL over time were related to brain volume loss but this correlation did not reach statistical significance (*r*_s_ = −0.324, *p* = 0.107). Changes in sNfL levels were unrelated to changes in WMH volumes. In stepwise linear regression analysis (Table 5, central row), we found that baseline normalized brain volume (*β* = 0.530, *p* < 0.01) was the strongest independent predictor explaining the development of brain atrophy. This was followed by change in sNfL but this again did not reach statistical significance (*β* = −0.334, *p* = 0.111).

In individuals ≥ 60 years of age (Table 4, right column) baseline sNfL correlated with baseline WMH volume (*r*_s_ = 0.141, *p* < 0.05) but not with baseline normalized brain volume. This correlation did not remain significant after correcting for age. Baseline sNfL was unrelated to changes in total brain volume and WMH volume over time. In stepwise linear regression analysis however, only baseline sNfL (*β* = −0.463, *p* < 0.001) and change in sNfL (*β* = −0.393, *p* < 0.01) could explain the development of brain atrophy over time (Table 5).

**Fig. 5:**
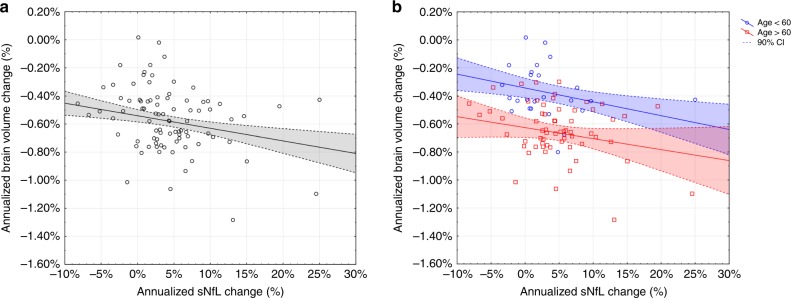
Brain atrophy and change in sNfL. Association between brain atrophy and change in sNfL per year in the entire cohort (**a**) (*r*_s_ = −0.290, *p* < 0.01) and divided in younger (age < 60 years = blue) and older subjects (age > 60 years = red) (**b**). In linear regression analyses, the annualized change in sNfL was identified as independent factor for the development of brain atrophy when considering the entire cohort (*β* = −0.336, *p* < 0.001) (**a**) and older individuals (>60 years) (*β* = −0.393, *p* < 0.01) (**b**). The dashed lines represent the 90% confidence interval. Two data points are outside the scaled range, but included in the analyses. Source data are provided as a Source Data file.

The association between brain volume change and change in sNfL per year and stratified by age is presented in Fig. 5.

### Association of sNfL and WMH with cognition

We then tested the association of sNfL with cognitive function (Mini-Mental State Examination (MMSE) score) in the entire cohort and across different age groups. Longitudinal analysis over more than 5 years revealed that in our cohort of neurological inconspicuous individuals, MMSE levels were rather stable during the follow-up period: Baseline MMSE: mean = 28.18, SD = ± 1.45, median = 28.0, IQR = 2.0; follow-up MMSE: mean = 28.03, SD = ± 1.56, median = 28.0, IQR = 2.0; and the annualized change in MMSE score was rather low: mean = −0.08, SD = ± 0.34, median = 0, IQR = 0.45. Applying the Wilcoxon signed-rank test to compare related baseline and follow-up values, we found a slight but still significant reduction in MMSE scores over time (*p* < 0.01). This difference was mainly driven by the age group above 60 years (*p* < 0.001) and was not significant analyzing individuals below 60 years.

Baseline sNfL correlated negatively with the annualized MMSE score change over time when considering the entire cohort (*r*_s_ = −0.273, *p* < 0.01) (Table 4). The annualized change in sNfL was unrelated to the annualized change in MMSE scores (Table 4, last row).

In addition, baseline WMH volume negatively correlated with the annualized MMSE score change over time in the entire cohort (*r*_s_ = −0.233, *p* < 0.05). The annualized change in WMH volume was unrelated to the annualized change in MMSE scores.

## Discussion

We here provide detailed description of the changes in sNfL levels in a normal population with aging both in a cross-sectional and longitudinal manner. sNfL was more stable in individuals below 60 years but thereafter considerably increased in a nonlinear manner. Likewise, and important for the use of sNfL in clinical practice, we observed a marked increase in the variability of sNfL levels with higher age particularly above 60 years. Furthermore, baseline sNfL was a strong and independent determinant of future brain volume loss and accelerated volume loss was paralleled by increases in sNfL concentration.

Normal aging is associated with neuronal loss and several attempts have been undertaken to accurately indicate this process, including morphologic^11^ and biochemical analyses^1^. With the advent of an ultra-high-sensitive NfL assay, it became feasible to study this marker for neuro-axonal damage in blood, which is an easy-to-access body fluid also allowing for repeated measurements. Although this has rapidly increased the number of reports on the role of sNfL in various neurological disorders, only scarce information is yet available regarding age-related ranges and variation of this marker in the normal aging population^1^. In CSF an age-related increase of NfL has been demonstrated in a cross-sectional study of 359 healthy individuals showing a linear correlation of log-transformed data (i.e. an exponential increase) with *r* = 0.77, *p* < 0.0001. The exponential yearly increase in CSF NfL was reported to be around 3.1%^12^. Another study on longitudinal CSF samples of 467 subjects, including individuals with subjective cognitive decline (*n* = 75), mild cognitive impairment (*n* = 128), Alzheimer’s disease dementia (*n* = 110), and cognitively unimpaired subjects (*n* = 154), reported a 4% yearly increase of NfL during a follow-up time of median of 2.1 years. Interestingly, this increase was similar among the investigated subgroups^13^.

Comparable results have been reported for blood NfL in a control group of 254 healthy individuals with a 2.2% increase per year on log-transformed data^4^. Although we here confirm an age-related increase in sNfL, our results demonstrate that the rise in sNfL with increasing age is nonlinear. Whereas sNfL seems to be more stable in individuals below 60 years, a considerable annualized increase of up to 4.3% is evident in individuals aged 60 years and older. This means that the maximum slope of annual change in sNfL in neurologically inconspicuous individuals may be reached starting already at the age of 60 years. This is biologically quite plausible, as the likelihood for concomitant or evolving, yet clinically silent, neurologic disorders is known to increase with age. In this context it is important to note that the observation of a nonlinear increase in NfL levels may be “masked” (Fig. 2b) when interpreting log-transformed data, an approach that has been followed in two previously mentioned studies for statistical reasons^4,12^.

Certainly, also other underlying mechanisms besides neuronal damage may contribute to an increase in sNfL with age. Thus, a reduced CSF turnover in the elderly may also factor in^14^. Nevertheless, the close relation between CSF and blood NfL levels suggests that both measures reflect similar pathophysiological processes^1,4,10^.

Other studies also reported an age-related increase of plasma NfL; one study included 193 cognitively healthy controls, 197 patients with a mild cognitive impairment and 180 patients with Alzheimer’s disease^15^ and another one included 41 nondemented controls, 25 patients with a mild cognitive impairment and 33 patients with Alzheimer’s disease^16^. Both studies, however, performed the correlation analysis on all three subgroups combinedly and they did not focus solely on the healthy control groups. An age-related increase in sNfL has further been reported in a group of 40 orthopedically injured patients who served as controls to study sNfL in 118 patients with a traumatic brain injury^17^. Of note, many of the 40 orthopedically injured patients had undergone brain MRI and some of them had preinjury neurological disease, including small infarcts, suspicion of glioma, etc. Furthermore, peripheral nerve injury could not be excluded, because neurophysiological examination has not been performed. These factors do therefore not allow direct comparison to our study cohort of neurologically inconspicuous individuals.

An important finding of our study is that the increase of sNfL in the age group above 60 years was paralleled by a substantial rise in variability of this marker. This has not been reported so far and suggests the contribution of subclinical brain tissue damage beyond the “normal” process of aging. Hence, we also sought for evidence that sNfL was associated with subclinical morphologic brain changes by brain MRI even in these clinically “normal” individuals. Our findings indeed showed that sNfL levels and its temporal dynamics are associated with brain volume loss over time. Although univariate analysis showed a significant correlation between sNfL levels and their temporal change with brain atrophy in the entire cohort, linear regression revealed this association was mainly driven by older individuals (>60 years). Less variability in sNfL seen in younger people probably due to a lower rate of concomitant non-age-related brain changes but also a lower number of included individuals below 60 years are likely explanations for this difference. On the other hand, the high variability in sNfL further demonstrates that also relatively low sNfL levels can be observed even in the older age groups. It is thus possible that levels in the low percentile groups in our cohort could reflect individuals with a healthier aging.

One recent study investigated cross-sectional CSF NfL levels in 144 cognitive healthy participants and followed them for 2 years clinically and with MRI. In a subset of 88 participants with full data available, it was demonstrated that CSF NfL levels independently predicted hippocampal atrophy. The age range of this cohort was 64−89 years; thus, there is still uncertainty if this association may also be present in younger people^18^.

In our normal aging cohort, we could not find any significant associations of sNfL with WMH volume, both by a cross-sectional and longitudinal analysis. This is in line with a recent study by our group where a correlation between sNfL and WMH score but not with WMH burden has been reported^19^ reflecting that the severity of tissue damage is probably more important than the volume of signal changes. Therefore, it is also not surprising that sNfL was positively correlated with WMH volume in CADASIL, suggesting that a certain threshold of WMH and extent of tissue destruction needs to be exceeded before sNfL may reflect such brain changes^19,20^.

We further tested if sNfL was associated with cognitive performance and we found that baseline sNfL levels correlated with the change in MMSE scores over time. At first sight, this appears somehow surprising, because in our normal aging cohort, MMSE scores remained rather stable over time, showing only a slight reduction during the follow-up period. However, sNfL appears to be sensitive enough to even indicate such subtle subsequent changes in cognitive function. In line with our findings and this assumption, other recent studies also found a relation of plasma or serum NfL with cognitive performance in controls^21,22^ and AD patients^16,21–23^.

In addition, we found that WMH volume correlated to reduced cognitive performance at baseline, but further change in WMH volume was unrelated to the change in MMSE scores. There are previous reports showing an association of WMH change with cognitive decline, but interestingly, this association was no longer significant when change in brain volume was added to the statistical models, indicating that cognitive decline related predominantly to the loss of brain volume with progression of lesion burden^24,25^.

At this point it is important to keep in mind that MMSE scores remained rather stable during the follow-up period with only little variance. This limits the potential to study determining factors of cognitive decline in our cohort. Future studies with longer follow-up duration and comprehensive cognitive data should be undertaken investigating such associations in more detail.

Highly sensitive blood NfL measurements may provide real-time information on the extent of neuro-axonal injury in various acute and chronic neurological disorders and may thus potentially aid in the clinical management of such patients. Although, at present, this measure may not discriminate between neuro-axonal injury, where neurons may potentially recover and permanent neuro-axonal loss, there is some evidence from smaller studies indicating that Nf increases in the extracellular fluid as a direct consequence of neuronal loss in patients with traumatic brain injury^26^. The process of ongoing neurodegeneration and neuro-axonal loss can also be captured at later stages using neuroimaging methods providing evidence for the development of brain atrophy. There now is an increasing body of evidence from longitudinal studies showing that higher sNfL levels are associated with development of brain atrophy (see, for example, refs. ^23,27^). These associations indicate a close relationship between higher sNfL levels and their changes over time and more pronounced brain atrophy as a result of ongoing axonal loss.

In this respect especially, the longitudinal aspect of sNfL measurements appears to be of interest and clinical utility as clear-cut levels of discrimination between normal and pathologic aging may be difficult to define especially in the elderly according to our findings. Therefore, we also concentrated specifically on the longitudinal changes of sNfL in our cohort. When using our PCTL model, we found that in 87% of individuals, baseline and follow-up sNfL levels remained within the same PCTL range, suggesting that in these individuals the observed yearly increase in sNfL was related to normal aging rather than any concomitant pathology. We hypothesize that care should be taken if follow-up sNfL levels switch to higher PCTL ranges, which may indicate the development of pathological processes; however, this certainly needs further confirmation in larger cohorts. The importance of evaluating repeated measurements of sNfL has also been demonstrated in a recent publication on presymptomatic inherited Alzheimer’s disease patients, showing that the dynamics of sNfL predicted neurodegeneration and clinical progression. The rate of change of sNfL could discriminate mutation carriers from nonmutation carriers more than 16 years before estimated symptom onset^23^. In a recent larger longitudinal study in older individuals (mean age > 70 years), plasma NfL concentrations were associated with imaging and cognition data in patients with Alzheimer’s disease (*n* at baseline = 327), mild cognitive impairment (*n* at baseline = 855) and in cognitively unimpaired subjects (*n* at baseline = 401). In the cognitively unimpaired control group, the rate of change in plasma NfL from baseline was lower (2.4 pg mL^−1^ per year) compared to MCI (2.7 pg mL^−1^ per year) and AD (4.9 pg mL^−1^ per year) and could further be related to imaging variables, including lower FDG-PET and increased atrophy measures^22^. Similarly, another longitudinal study with a median follow-up time of 15 or 30 months analyzed CSF and plasma NfL levels in 79 elderly (median age 76.4 years), including 15 subjects with a mild cognitive impairment. Higher baseline NfL levels were associated with worsening in neuroimaging measures and global cognition and the change in plasma NfL was associated with change in global cognition, attention, and amyloid PET^21^. However, both studies included only elderly individuals and investigated NfL changes from baseline and therefore no information on the evolution of NfL across different age ranges is provided.

The present study has several limitations. First, our cohort does not entirely cover all age ranges as we investigated a population aged between 38 and 85 years and therefore information especially regarding younger adults is lacking. Second, the number of individuals in younger age groups was partly quite low, which may have limited statistical power. Third, present findings are derived from a single-center cohort and future studies are therefore needed to validate this marker in a multicenter setting. Fourth, other markers have been examined in the context of normal brain aging, including serum S100β^28^ and future studies should clarify the role and relevance of these and additional biomarkers also in combination. Finally, unfortunately we do not have any information on amyloid PET and CSF biomarkers in our cohort. Although this would have been interesting, particularly to compare our results to CSF Alzheimer’s disease biomarkers, in the Austrian Stroke Prevention Study, lumbar puncture was not included in the study protocol. Present data provide detailed longitudinal characterization of age-related changes in sNfL together with imaging findings in a normal neurological inconspicuous population, showing a nonlinear increase with age and a close association with brain volume loss. This information is fundamental in order to correctly interpret sNfL values in various neurological disorders.

## Methods

### Participants

Our investigation was based on data and material from the prospective and ongoing Austrian Stroke Prevention Family Study (ASPS-Fam), which is an extension of the Austrian Stroke Prevention Study (ASPS) that was established in 1991 ^8,9^. Between 2006 and 2013, study participants of the ASPS and their first-grade relatives were invited to enter ASPS-Fam. Inclusion criteria were no history of stroke or dementia and a normal neurological examination. None of the subjects had suffered a traumatic brain injury. The entire cohort underwent a thorough diagnostic work-up including clinical history taking, laboratory evaluation, cognitive testing, and an extended vascular risk factor assessment. Thus far the ASPS-Fam study comprises of 381 individuals from 169 families with MRI and blood samples available in 371 participants. Another 36 subjects had to be excluded due to one or more of following exclusion criteria: diagnosis or suspicion of dementia (MMSE ≤ 24 or problems (failure of one task in the Mini-Cog test) of memory: *n* = 11), visible brain infarcts on MRI (*n* = 19), a history of stroke (*n* = 9), other diseases (chronic myeloid leukemia) (*n* = 1). This left a total of 335 participants to investigate sNfL in an aging population.

From all subjects, 103 agreed on a follow-up scan and the mean follow-up time was 5.59 years (SD = ± 0.97, min = 3.99, max = 6.94). We applied the same inclusion and exclusion criteria at the follow-up visit as for the baseline visit. A total of eight follow-up cases were excluded for new onset stroke (*n* = 3), heart disease (*n* = 2), transient ischemic attack (*n* = 1), brain hemorrhage (*n* = 1) and orofacial dyskinesia (*n* = 1) between baseline and follow-up assessment. Therefore, the final longitudinal dataset consisted of 95 individuals.

Due to motion artifacts or compliance of the subjects, the number of subjects in the following variables was reduced: brain volume (*n* = 330), brain volume change (*n* = 89), WMH volume (*n* = 320), WMH volume change (*n* = 88), MMSE change (*n* = 94).

The study protocol was approved by the ethics committee of the Medical University of Graz, Austria, and written informed consent was obtained from all participants.

### Magnetic resonance imaging

Magnetic resonance imaging was performed on a 3T whole-body MR system (TimTrio; Siemens Healthcare, Erlangen, Germany) with a 12-channel head coil. To assess morphologic brain changes, we analyzed the brain volume and the volume of WMH as the most common age-related cerebral abnormality^9^ in a cross-sectional and longitudinal manner.

Brain volume measurements, normalized by subject head size, were performed on a high-resolution T1-weighted 3D sequence with magnetization prepared rapid gradient echo (MPRAGE) with whole-brain coverage (TR = 1900 ms, TE = 2.19 ms, TI = 900 ms, flip angle = 9°, isotropic resolution of 1 mm). We used SIENAX to calculate cross-sectional data and longitudinal brain atrophy was estimated from two time points with SIENA, both programs being part of FSL^29,30^.

The scan protocol also included an axial FLAIR sequence (TR = 10,000 ms, TE = 69 ms, TI = 2500 ms, number of slices = 40, resolution = 0.86 × 0.86 × 3 mm), on which a trained rater identified WMH slice by slice using the semi-automatic program DISPIMAGE^31,32^.

### Blood samples

Serum samples were collected by venipuncture and processed on the same day within 2 h. After venipuncture, blood tubes remained at room temperature for 30 min and were then centrifuged at room temperature for 10 min at 2000 × *g*. Serum was then aliquoted in polypropylene tubes and stored at −70 °C until analysis.

### NfL measurements

All serum samples were analyzed at the University Hospital Basel, Switzerland. Serum NfL levels were determined by single molecule array (Simoa) assay using the capture monoclonal antibody (mAB) 47:3 (initial dilution 0.3 mg/mL; Art. No. 27016) and the biotinylated detector mAB 2:1 (0.1 μg/mL; Art. No. 27018) from UmanDiagnostic^33^ transferred onto the Simoa platform^4^. The samples from the same participants were analyzed together in the same run to avoid within-subjects run-to-run variability. Intra- and interassay variability of the measurements were evaluated with three native serum samples in five consecutive runs on independent days. The mean coefficients of variation (CVs) of duplicate determinations for concentration were 8.5% (9.5 pg mL^−1^, sample 1), 5.4% (23.2 pg mL^−1^, sample 2) and 7.8% (98.5 pg mL^−1^, sample 3). Interassay CVs for serum were 7.8% (sample 1), 8.3% (sample 2) and 4.9% (sample 3).

### Statistical analysis

Statistical analysis was performed using the statistical software R (version 3.5.2; R: a language and environment for statistical computing, Vienna, Austria) and SPSS (version 20; SPSS Inc., Chicago, IL). A two-sided *p* value (*p*) < 0.05 was considered to be statistically significant. To test the assumption for normal distribution, we used the Shapiro–Wilk test. Normally distributed variables are reported as mean ± SD and non-normally distributed variables as median and the 25th and 75th quartile. In order to test, whether sNfL differs between gender, we applied the nonparametric Mann−Whitney *U* test for each age category as well as the entire cohort. For correlation analyses we used Spearman correlation for non-normally distributed and Pearson correlation for normally distributed data.

The development of sNfL levels through the lifespan was analyzed by building age categories with a range of 10 years (<50, 50−60, 60−70, >70). We calculated levels of sNfL at following percentiles (PCTL) (25th, median, 65th, 80th, 90th and 95th PCTL) in each age category and visualized their progression by a color scale, reaching from the lowest PCTL range (light-yellow = 0−25th PCTL) to the highest (red = 90th−95th PCTL). The specific ranges were selected based on visual inspection of the spread of sNfL values.

The corresponding visualization (Fig. 1) was obtained by a spline interpolation of the sNfL levels in each PCTL range over the age (smoothing factor = 0.03, age−range = 38−88, resolution-steps = 1 year).

To find independent determinants of brain atrophy, we used a backward stepwise regression model, which starts with all exploratory variables and removes the least significant in each step. To assess the equality of variances of sNfL in each age category, we performed the Brown−Forsythe test, which uses the median in each category to measure the spread in each group with the benefit to provide robustness against non-normal distributed data. To account for the relatedness between the study participants in the current family-based study, regression analysis was performed using linear mixed models with a random effect, which describes the family structure by means of a kinship matrix^34,35^.

### Reporting summary

Further information on research design is available in the Nature Research Reporting Summary linked to this article.

## Supplementary information

Reporting Summary

## Data Availability

The source data of key findings underlying Figs. 1, 2, 3, 4, 5 and Tables 2, 3, 4, 5 are provided as Source Data file. All other data sets generated during and/or analyzed during the current study are available from the corresponding author upon reasonable request.

## Source data

Source Data
