# Supplementary material for: Serum neurofilament light levels in normal aging and their association with morphologic brain changes
Source: Nat Commun. 2020 Feb 10;11:812. doi: 10.1038/s41467-020-14612-6 (PMC7010701; doi:10.1038/s41467-020-14612-6)
Supplement: Supplementary file 1 — Reporting Summary [file 41467_2020_14612_MOESM1_ESM.pdf]

nature research

Corresponding author(s): Dr. Michael Khalil

Last updated by author(s): Jan 3, 2020

Reporting Summary

Nature Research wishes to improve the reproducibility of the work that we publish. This form provides structure for consistency and transparency in reporting. For further information on Nature Research policies, see Authors & Referees and the Editorial Policy Checklist.

Statistics

For all statistical analyses, confirm that the following items are present in the figure legend, table legend, main text, or Methods section.

n/a	Confirmed	
☐	☒	The exact sample size (n) for each experimental group/condition, given as a discrete number and unit of measurement
☐	☒	A statement on whether measurements were taken from distinct samples or whether the same sample was measured repeatedly
☐	☒	The statistical test(s) used AND whether they are one- or two-sided<br>*Only common tests should be described solely by name; describe more complex techniques in the Methods section.*
☐	☒	A description of all covariates tested
☐	☒	A description of any assumptions or corrections, such as tests of normality and adjustment for multiple comparisons
☐	☒	A full description of the statistical parameters including central tendency (e.g. means) or other basic estimates (e.g. regression coefficient) AND variation (e.g. standard deviation) or associated estimates of uncertainty (e.g. confidence intervals)
☒	☐	For null hypothesis testing, the test statistic (e.g. F, t, r) with confidence intervals, effect sizes, degrees of freedom and P value noted<br>*Give P values as exact values whenever suitable.*
☒	☐	For Bayesian analysis, information on the choice of priors and Markov chain Monte Carlo settings
☒	☐	For hierarchical and complex designs, identification of the appropriate level for tests and full reporting of outcomes
☒	☐	Estimates of effect sizes (e.g. Cohen's d, Pearson's r), indicating how they were calculated

Our web collection on statistics for biologists contains articles on many of the points above.

Software and code

Policy information about availability of computer code

Data collection	FSL-Tools (fsl.fmrib.ox.ac.uk), DispImage (D.L. Plummer 1992)
Data analysis	R-Foundation for Statistical Computing, IBM SPSS Statistics (v25.0), MATLAB

For manuscripts utilizing custom algorithms or software that are central to the research but not yet described in published literature, software must be made available to editors/reviewers. We strongly encourage code deposition in a community repository (e.g. GitHub). See the Nature Research guidelines for submitting code & software for further information.

Data

Policy information about availability of data

All manuscripts must include a data availability statement. This statement should provide the following information, where applicable:

- Accession codes, unique identifiers, or web links for publicly available datasets
- A list of figures that have associated raw data
- A description of any restrictions on data availability

The source data of key findings underlying Figs. 1, 2, 3, 4, 5 and Tables 2, 3, 4 are provided as Source Data file. All other data sets generated during and/or analyzed during the current study are available from the corresponding author upon reasonable request.

Field-specific reporting

Please select the one below that is the best fit for your research. If you are not sure, read the appropriate sections before making your selection.

☒ Life sciences ☐ Behavioural & social sciences ☐ Ecological, evolutionary & environmental sciences

Life sciences study design

All studies must disclose on these points even when the disclosure is negative.

Sample size	Our investigation was based on data and material from the prospective and ongoing Austrian Stroke Prevention Family Study (ASPS-Fam), which is an extension of the Austrian Stroke Prevention Study (ASPS) that was established in 1991 (Schmidt, R. et al. Assessment of cerebrovascular risk profiles in healthy persons: definition of research goals and the Austrian Stroke Prevention Study (ASPS). Neuroepidemiology 13, 308–13 (1994); Schmidt, R., Fazekas, F., Kapeller, P., Schmidt, H. & Hartung, H. P. MRI white matter hyperintensities: three-year follow-up of the Austrian Stroke Prevention Study. Neurology 53, 132–9 (1999)). Between 2006 and 2013, study participants of the ASPS and their first-grade relatives were invited to enter ASPS-Fam. Inclusion criteria were no history of stroke or dementia and a normal neurological examination. The entire cohort underwent a thorough diagnostic work-up including clinical history taking, laboratory evaluation, cognitive testing, and an extended vascular risk factor assessment. Thus far the ASPS-Fam study comprises of 381 individuals from 169 families with MRI and blood samples available in 371 participants.<br>From all subjects, 103 agreed on a follow-up scan and the mean follow-up time was 5.59 years (±0.97, min=3.99, max=6.94). A total of 8 follow-up cases were excluded for new onset stroke (n=3), heart disease (n=2), transient ischemic attack (n=1), brain hemorrhage (n=1) and orofacial dyskinesia (n=1) between baseline and follow-up assessment.
Data exclusions	Thirty-six subjects had to be excluded due to one or more of following exclusion criteria: diagnosis or suspicion of dementia (MMSE<=24 or problems (failure of one task in the Mini-Cog test) of memory: n=11), visible brain infarcts on MRI (n=19), a history of stroke (n=9), other diseases (chronic myeloid leukemia) (n=1). This left a total of 335 participants to investigate sNfL in an aging population.<br>From all subjects, 103 agreed on a follow-up scan and the mean follow-up time was 5.59 years (±0.97, min=3.99, max=6.94). A total of 8 follow-up cases were excluded for new onset stroke (n=3), heart disease (n=2), transient ischemic attack (n=1), brain hemorrhage (n=1) and orofacial dyskinesia (n=1) between baseline and follow-up assessment.
Replication	From the 335 included participants, 103 agreed on a follow-up scan and the mean follow-up time was 5.59 years
Randomization	Randoimzation was not relevant for this study.
Blinding	All analysis have been performed by trained coworkers, blindend to clinical informtion.

Reporting for specific materials, systems and methods

We require information from authors about some types of materials, experimental systems and methods used in many studies. Here, indicate whether each material, system or method listed is relevant to your study. If you are not sure if a list item applies to your research, read the appropriate section before selecting a response.

Materials & experimental systems

n/a	Involved in the study	
☐	☒	Antibodies
☒	☐	Eukaryotic cell lines
☒	☐	Palaeontology
☒	☐	Animals and other organisms
☐	☒	Human research participants
☐	☒	Clinical data

Methods

n/a	Involved in the study	
☒	☐	ChIP-seq
☒	☐	Flow cytometry
☐	☒	MRI-based neuroimaging

Antibodies

Antibodies used	All serum samples were analyzed at the University Hospital Basel, Switzerland by board certified technicians blinded to clinical or paraclinical information. Serum NfL levels were determined by single molecule array (Simoa) assay using the capture monoclonal antibody (mAB) 47:3 and the biotinylated detector mAB 2:1 from UmanDiagnostic (Norgren, N., Karlsson, J.-E., Rosengren, L. & Stigbrand, T. Monoclonal antibodies selective for low molecular weight neurofilaments. Hybrid. Hybridomics 21, 53–9 (2002)) transferred onto the Simoa platform as previously described (Disanto, G. et al. Serum Neurofilament light: A biomarker of neuronal damage in multiple sclerosis. Ann. Neurol. 81, 857–870 (2017)).
Validation	Recovery rates ([concentration of spiked sample - concentration of native sample]/spiked concentration x 100) were tested in 4 serum and 4 CSF samples from HC spiked with 5, 50, and 200pg/ml and 500 and 2,000pg/ml of NfL, respectively. The mean recovery after spiking was 107% for serum and 121% for CSF. Parallelism and linearity of the assay for serum and CSF were confirmed by serial dilution experiments (Disanto, G. et al. Serum Neurofilament light: A biomarker of neuronal damage in multiple sclerosis. Ann. Neurol. 81, 857–870 (2017); Valentin, MA. et al. Validation of immunoassay for protein biomarkers: bioanalytical study plan implementation to support pre-clinical and clinical studies. J Pharm Biomed Anal. 55, 869–877 (2011). The used monoclonal antibodies have proven to be highly specific in NfL knock-out animal experiments (Bacioglu, M. et al. Neurofilament light chain in blood and CSF as marker of disease progression in mouse models and in neurodegenerative diseases. Neuron. 91, 56-66 (2016).

Human research participants

Policy information about studies involving human research participants

Population characteristics	We analyzed participants from the prospective and ongoing Austrian Stroke Prevention Family Study (ASPS-Fam), which is an extension of the Austrian Stroke Prevention Study (ASPS) that was established in 1991 (see above).<br>Mean age was 64.85 years, and the cohort consisted of 195 female and 140 male individuals.
Recruitment	Participants were randomly recruited based on the official registry of residents of city of Graz, Austria, between 1991 and 1994. Between 2006 and 2013, study participants of the ASPS and their first-grade relatives were invited to enter ASPS-Fam.
Ethics oversight	The study protocol was approved by the ethics committee of the Medical University of Graz, Austria, and written informed consent was obtained from all participants.

Note that full information on the approval of the study protocol must also be provided in the manuscript.

Clinical data

Policy information about clinical studies

All manuscripts should comply with the ICMJE guidelines for publication of clinical research and a completed CONSORT checklist must be included with all submissions.

Clinical trial registration	Retrospective analysis of the ASPS-Family, supported by Austrian Science Fund P20545-P05, P13180 and I904-B13 (Era-Net)
Study protocol	MRI, cognitive testing, and cardiovascular risk factor assessment
Data collection	Data used for the current study were collected between 2006 and 2013
Outcomes	*Describe how you pre-defined primary and secondary outcome measures and how you assessed these measures.*

Magnetic resonance imaging

Experimental design

Design type	Structural analysis and visual rating
Design specifications	-
Behavioral performance measures	-

Acquisition

Imaging type(s)	Structural
Field strength	3T
Sequence & imaging parameters	T1 MPRAGE at 1mm isotropic in-plane resolution (TR=1900ms, TE=2.19ms, TI=900ms, FA=9°), axial T2w-FLAIR (TR=10s,TE=69ms,TI=2500ms, number of slices=40, resolution=0.86x0.86x3mm)
Area of acquisition	whole brain

Diffusion MRI ☐ Used ☒ Not used

Preprocessing

Preprocessing software	No specific preprocessing was used. For SIENA/X neck-cutting was applied.
Normalization	No specific normalization was applied.
Normalization template	SIENAX uses an affine-registration to the MNI152 template in order to normalise for head size
Noise and artifact removal	visual inspection to exclude scans with motion artifacts
Volume censoring	*Define your software and/or method and criteria for volume censoring, and state the extent of such censoring.*

Statistical modeling & inference

Model type and settings	-
Effect(s) tested	-

Specify type of analysis: ☒ Whole brain ☐ ROI-based ☐ Both

Statistic type for inference<br>(See Eklund et al. 2016)	-
Correction	-

Models & analysis

n/a	Involved in the study
☒	☐ Functional and/or effective connectivity
☒	☐ Graph analysis
☒	☐ Multivariate modeling or predictive analysis
